# Selenium Content in Staple Crops and Drinking Water and Associated Health Risk Assessment: A Case Study of a Selenium-Rich Region in Southern Shaanxi, China

**DOI:** 10.3390/foods15010031

**Published:** 2025-12-22

**Authors:** Yangchun Han, Litao Hao, Shixi Zhang, Kunli Luo, Lijun Zhang, Weiqiang Chen

**Affiliations:** 1 School of Geosciences and Surveying Engineering, China University of Mining and Technology Beijing, Beijing 100083, China; hanycn@foxmail.com; 2 Office of the State-Owned Assets Committee, Hebei Normal University, Shijiazhuang 050024, China; 3 Institute of Geographic Sciences and Natural Resources Research, CAS, Beijing 100101, China; 4 Ankang R&D Center for Se-Enriched Products, China Se-Enriched Industry Research Institute, Ankang 725000, China; 5 Shaanxi Xibao Technology Co., Ltd., Ankang 725000, China

**Keywords:** excessive-Se, high-Se, drinking water–crop systems, daily intake, health risks, children, adults

## Abstract

To study the selenium (Se) content and dietary risk in typical Se-rich regions (the soil Se thresholds were as follows: high Se at 0.4–3.0 mg/kg and excessive Se at >3.0 mg/kg), the northern of Langao County, Ankang City, Shaanxi Province was studied. Contents of Se in crops and drinking water were analyzed. The average Se contents in drinking water was 12.32 μg/L in the excessive-Se and 13.50 μg/L in the high-Se areas. Corn, rice, sweet potato, and eggplant exhibited the highest average Se contents in the excessive-Se area, while potato and radish showed the highest levels in the high-Se area. Adults (and children) living in excessive-Se areas had a mean daily Se intake of 598 (305) μg/day, and those in high-Se areas had an intake of 536 (275) μg/day. Although crops were the main dietary source of Se, the contribution of drinking water, particularly for children, should not be overlooked as an additional source of Se intake. The average hazard quotients of adults (children) from excessive-Se and high-Se areas were 1.77 (1.95) and 1.58 (1.76), respectively. Therefore, there are non-carcinogenic health risks for humans in the two regions.

## 1. Introduction

Selenium (Se) is an essential trace element that promotes human growth and development [1]. Both excessive and inadequate Se intake can pose risks to human health [2,3]. Excessive Se consumption can lead to Se poisoning symptoms such as hair and nail loss [4,5]. Inadequate Se consumption can result in Keshan disease (KD) or Kashin–Beck disease (KBD) [1,6].

Human (low seafood-consuming communities) intake of Se from the environment is primarily through drinking water and consuming food crops. Hence, the Se content in the environment is of widespread concern [7,8,9]. Approximately 72% of China’s land area is not rich in Se, with soil Se content below 0.4 mg/kg [10]. Cases of KD and KBD caused by Se deficiency have been found in 329 counties across 16 provinces of China [1,6]. Based on the results of epidemiological surveys, Tan Jian’an (1989) defines Se-rich regions as areas with soil Se content greater than 0.4 mg/kg and can be further divided into high-Se areas (0.4–3.0 mg/kg) and excessive-Se areas (>3.0 mg/kg) [11].

Nowadays, Se-rich regions are actively developing Se-enriched products which are sold nationwide [12]. Therefore, studies on the health risk associated with Se in drinking water and crops in Se-rich regions is of great importance. Many studies have focused on the daily intake of Se and the associated health risks within the drinking water–crop system in Se-rich regions, both domestically and internationally. For example, the daily intake of Se through drinking water for residents in the Se-rich central region of Punjab, India, was 0.02–71.1 μg/day (mean 1.73 μg/day), and the hazard quotient (HQ) was 0.34–0.60 (mean 0.35) [13]. The daily intake of Se through dietary consumption by residents in the Se-rich Enshi district of China was 1067 μg/day, and the HQ was 4.34 [4]. In brief, previous studies have predominantly treated Se-rich regions as a unified entity. However, the distribution of soil Se content in Se-rich regions is uneven [2,14]. This disparity can lead to significant variations in Se levels in crops and drinking water, ultimately failing to accurately reflect the true Se intake and health risks for residents of these regions. Additionally, plenty of studies have predominantly focused on excessive-Se areas (soil Se >3.0 mg/kg), which present a significantly elevated risk of Se toxicity [4,15,16]. In contrast, high-Se areas (with soil Se 0.4–3.0 mg/kg), which were generally considered safe and more suitable for long-term human habitation, have received less attention [17]. The dietary safety of long-term residency in high-Se areas for residents, as well as whether there are differences in Se content (in crops and drinking water) and the consequent health effects for humans between high-Se and excessive-Se areas, remain unclear.

This study investigates the Se content in drinking water–crop systems, estimates daily intake, and assesses health risks for adults and children in typical excessive-Se and high-Se areas. A total of 39 drinking water samples and 97 crops were collected in Zuolong Town and Minzhu Town in Langao County, Shanxi Province, China. The main objectives were as follows: (1) determine the Se content in drinking water and crops within excessive-Se and high-Se areas; (2) estimate daily Se intake for adults and children in each area; and (3) assess potential health risks associated with Se intake in adults and children in each area. This study aims to provide scientific evidence for guiding residents in Se-rich regions toward making dietary choices for safe Se intake.

## 2. Materials and Methods

### 2.1. Study Area

**Figure 1:**
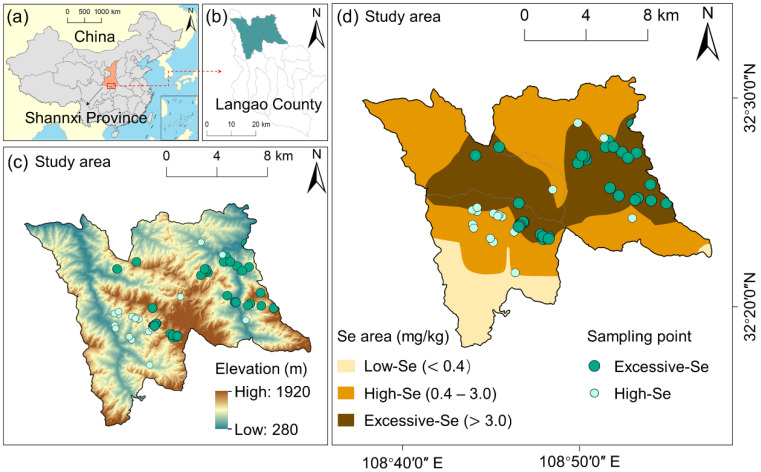
Location and description of the study area: (**a**) location of Shaanxi Province in China; (**b**) location of study area in Langao County; (**c**) elevation map; (**d**) spatial distribution of soil Se concentration and sampling sites.

The study area encompasses Zuolong Town and Minzhu Town in the northern part of Langao County, Shaanxi Province, covering 18% of the county’s total area (348.49 km^2^) (Figure 1). Within this area, soils with excessive Se content (Se > 3 mg/kg) account for 29.52%, while soils with high Se content (0.4–3 mg/kg) cover 56.56% of the study area [18]. The region is situated in the hinterland of the Daba Mountains, characterized by predominantly mountainous terrain with a general trend of higher elevations to the south and lower elevations to the north (309–1941 m above sea level). The climate of the study area falls within the subtropical humid monsoon climate category, featuring abundant rainfall and distinct seasons, with an average annual temperature of 16 °C. Annual average precipitation measures at 1100 mm, with rainfall primarily concentrated between July and September each year. The rivers within this area belong to the Hanjiang River system in the Yangtze River basin, with major rivers including the Dadao River and Lanhe River. The predominant exposed geological strata in this region are early Paleozoic formations, notably dominated by Cambrian strata associated with a high-Se geological background [8,19].

The mountainous areas of Zuolong Town and Minzhu Town in the northern part of Langao County are characterized by deep valleys and Se-rich soil. Spring water is extensively distributed and commonly consumed by residents. The study area is situated in a remote mountainous region, where inconvenient transportation due to natural and geographical constraints has led to an economy primarily reliant on agriculture and eco-tourism, resulting in relatively slow development [8,19]. Consequently, residents mainly follow a low-protein diet with very limited intake of meat, eggs, and dairy [20,21]. In 2018–2020, through a field survey of Langao County, it was found that several kinds of crops, including corn, rice, sweet potato, potato, radish, and eggplant, are most commonly cultivated and consumed by local residents. Although no prior references support these findings, we hope our survey results will contribute valuable data for future research in this region.

### 2.2. Sampling and Processing

From 2018 to 2020, a total of 136 samples were collected from the study area, including spring water (*n* = 39), corn (*n* = 34), rice (*n* = 12), sweet potato (*n* = 12), potato (*n* = 18), radish (*n* = 10), and eggplant (*n* = 11). All samples were collected from households or in the fields of rural areas located far away from urban centers and industrial or mining enterprises. The crop samples were collected from the edible components of indigenous plants that have not undergone any form of processing or treatment. Prior to analysis, all crops underwent triple washing with ultra-pure water. Corn and rice were subsequently dried at 60 °C in an oven, while sweet potato, potato, radish, and eggplant were blanched before being dried at the same temperature. The dried crops were then pulverized into powder using a high-speed universal mill (Tianjin Test Instrument Co., Ltd., Tianjin, China, FW80) and stored in plastic bottles at 4 °C for testing purposes [22,23]. The collection of spring water (as drinking water) followed standardized procedures, which included washing polyethylene bottles three times with raw water, collecting flowing water samples, and storing them at 4 °C until analysis [24,25,26]. All samples were processed from collection to testing within one week.

### 2.3. Chemical Analysis

Analysis of the samples was conducted at the Analytical Testing Center of the Institute of Geographic Sciences and Natural Resources Research, Chinese Academy of Sciences. Se content in water samples was determined using a tandem inductively coupled plasma mass spectrometer (ICP-MS/MS, 8900, Agilent, Santa Clara, CA, USA), while the Se content in crops was analyzed using hydride generation–atomic fluorescence spectrometry (HG-AFS, AFS-9780, Haiguang, Beijing, China) [23,27]. The operating parameters for the ICP-MS/MS and HG-AFS instruments are shown in Tables S1 and S2. The primary objective of this study was to conduct a geographical survey to assess the overall Se exposure level and its association with dietary intake in the study area. While we acknowledge that Se speciation (e.g., selenomethionine and selenate) influences its bioavailability and toxicity, the measurement of total Se concentration serves as the fundamental and critical first step for identifying Se deficiency or excess. Therefore, our methodology focused on total Se analysis to provide a baseline for future, more targeted speciation studies.

### 2.4. Evaluation Models

#### 2.4.1. Estimation of Daily Intake Levels

The daily intake combination of residents (including adults and children) is generally grain, vegetables, and drinking water. Considering that residents of Langao County are self-sufficient in terms of production and lifestyle, the crops and spring water collected in this study can accurately reflect the Se intake levels of residents in excessive-Se or high-Se areas based on specific dietary combinations [19,21]. In addition, in order to maximize the reflection of residents’ daily Se intake, it was assumed that individuals residing in excessive-Se or high-Se areas consume only one type of grain and vegetable along with drinking water on a daily basis. Specifically, the daily intakes of Se based on nine different dietary grain–vegetable–drinking water combinations (corn–potato–water, corn–radish–water, corn–eggplant–water, rice–potato–water, rice–radish–water, rice–eggplant–water, sweet potato–potato–water, sweet potato–radish–water, and sweet potato–eggplant–water) were estimated in the areas of excessive-Se and high-Se, respectively. The estimated daily intake (EDI) of residents was calculated using Equation (1) [14]:

(1) $$EDI=C_{grain}\times {IR}_{grain}+C_{vegetable}\times {IR}_{vegetable}+C_{water}\times {IR}_{water}$$

where the EDI was the daily dietary Se intake (μg/day); C_grain_, C_vegetable_, and C_water_ were the Se concentrations (μg/kg) of grains, vegetables, and drinking water, respectively; and IR_grain_ and IR_vegetable_ refer to the average daily consumption of adults in Shaanxi Province in 2021 (grains: 0.421 kg/day; vegetables: 0.276 kg/day), sourced from the National Bureau of Statistics (https://data.stats.gov.cn/). Children’s daily consumption of crops was half that of adults; IR_water_ was the daily intake rate of drinking water (2 L/day for adults and 1.5 L/day for children) [28]. The target food items were categorized into three groups: grains (rice, corn, and sweet potato), vegetables (potato, radish, and eggplant), and water (drinking water). Although the diversity of dietary habits of residents does exist, this paper studies the maximum dietary health tolerance of residents under specific dietary patterns based on the background of a typical Se-rich region. This approach allows us to estimate Se intake from a single high-Se crop, thus identifying the maximum safe consumption levels of each crop based on Se limits, which provides valuable dietary guidance for residents’ health.

#### 2.4.2. Health Risk Assessment Model

The HQ proposed by the United States Environmental Protection Agency (USEPA) has been widely and successfully used to evaluate the potential non-carcinogenic risks that trace elements may pose to human health through skin, inhalation, and oral routes [7,28]. Because oral intake represented the most significant route of Se absorption for the human body, the health risk assessment model proposed by the USEPA was used to evaluate the health risks caused by the consumption of drinking water and crops for humans (including adults and children) [28,29]. The HQ, defined as the ratio of the chronic daily intake (CDI) to the reference dose (RfD), was calculated by the theoretical model as the evaluation standard. When HQ > 1, it indicates that the consumer group may be at risk of Se exposure due to the consumption of drinking water–crops; when HQ ≤ 1, it indicates that the consumer group was not at risk of Se exposure. The smaller the HQ value, the lower the exposure risk [28]. Table 1 presents the parameters and definitions of the health risk assessment model. The value of HQ was calculated using Equations (2) and (3):

(2) $$CDI=\frac{EDI\times EF\times ED}{BW\times AT}$$

(3) $$HQ=\frac{CDI}{RfD}$$

### 2.5. Quality Control and Statistical Analysis

Two (two replicates) certified reference materials [GBW10012 (corn), GBW10010a (rice)] were used for the quality control of every batch of crop samples [22,23]. The recovery of Se in the Chinese standard reference substances (rice and corn) was between 90% and 110%, all within the range of certified values, indicating that the whole determination process was accurate and reliable (Table S3). The calibration ranges, R-squared values, detection limits (3σ/k), and quantification limits (10σ/k) for the calibration curves are listed in Table S4 (σ: the standard deviation of 11 replicates of blank measurements; k: the slope of the calibration line). Two sets of blank samples were included in each batch of tested samples, and one sample was retested every 15 samples, with the relative error of the samples falling within ±10% [19,30]. The software utilized in this study included ArcGIS 10.2, Origin 2021, and Excel 2016.

## 3. Results and Discussion

### 3.1. Content of Se in Drinking Water

The content of Se in 23 spring water (as drinking water) samples from an excessive-Se area and 16 spring water samples from a high-Se area was studied. The average Se content in spring water from the excessive-Se area (*n* = 23) was 12.32 μg/L (0.31–30.52 μg/L). All spring water samples have Se content lower than the world drinking water limit standard (40 μg/L), while 16 spring water samples have Se content higher than the Chinese drinking water limit standard (10 μg/L) [31,32]. The Se content in spring water samples from excessive-Se areas varied greatly, with a coefficient of variation of 57.08% (Table S5). The average Se content in spring water from the high-Se area (*n* = 16) was 13.50 μg/L (0.55–41.19 μg/L). A total of 15 spring water samples have Se content lower than the world drinking water limit (40 μg/L), while 10 spring water samples have Se content higher than the Chinese drinking water limit (10 μg/L) [31,32]. The Se content in spring water samples from high-Se areas varied greatly, with a coefficient of variation of 86.75% (Table S5).

It was noteworthy that 16 of the 23 spring water samples collected from an excessive-Se area (with a Se enrichment rate of 69.57%) met the standard of Se-rich mineral water [33]. Similarly, 10 of the 16 spring water samples collected from a high-Se area (Se enrichment rate 62.50%) met the standard of Se-rich mineral water [33]. The average Se content in spring water samples from excessive-Se and high-Se areas was higher than that in high-end mineral water from Italy (0.26 μg/L) and Switzerland (0.25 μg/L) in Europe [34,35].

### 3.2. Content of Se in Crops

**Figure 2:**
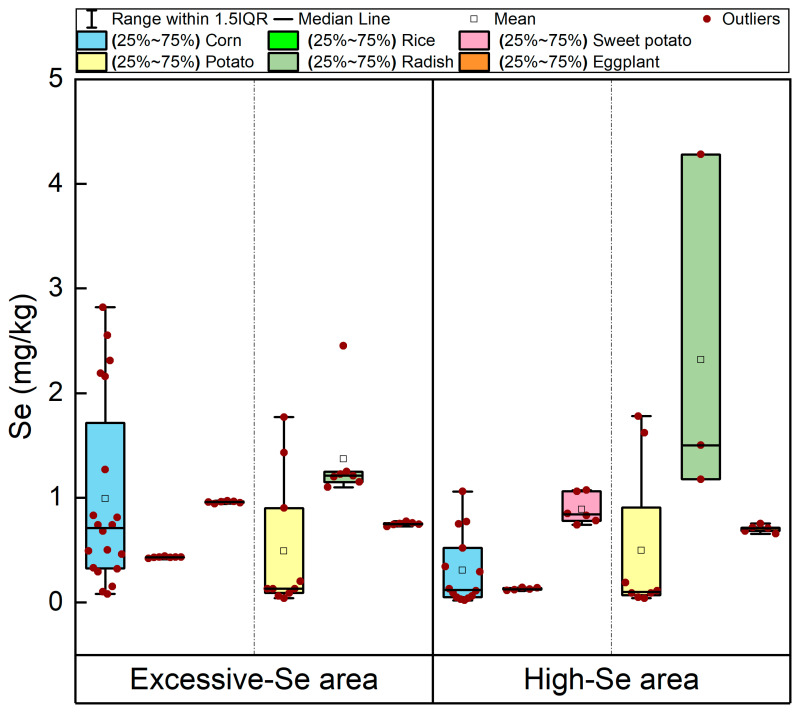
Se content in crops of excessive-Se and high-Se areas.

The contents of Se in crops from excessive-Se and high-Se areas are shown in Figure 2. Crops exhibited peak Se contents of 2.82 mg/kg in corn, 0.44 mg/kg in rice, 1.07 mg/kg in sweet potato, 1.78 mg/kg in potato, 4.28 mg/kg in radish, and 0.77 mg/kg in eggplant, which were 8.8 to 214 times the local Se standard set for the Shaanxi Province [36]. The crops cultivated in the excessive-Se area showed mean Se content in the following descending order: radish (1.37 mg/kg), corn (0.99 mg/kg), sweet potato (0.96 mg/kg), eggplant (0.75 mg/kg), potato (0.49 mg/kg), and rice (0.43 mg/kg). The crops cultivated in the high-Se area showed mean Se content in the following descending order: radish (2.32 mg/kg), sweet potato (0.89 mg/kg), eggplant (0.70 mg/kg), potato (0.50 mg/kg), corn (0.30 mg/kg), and rice (0.13 mg/kg). Interestingly, radish had the highest mean Se content in the two distribution areas, while rice had the lowest average Se content, resulting in a maximum difference of 3.19 times and 17.85 times between them (Table S6). This result suggests that the Se content of rhizome (radish) was higher than that of grains (rice), which might be attributed to the greater absorption capacity of plant roots for Se compared to its transport capacity within the plant [37,38].

The Se enrichment rate (i.e., the proportion of samples that meet the Se enrichment standard out of all samples) for crops was 100% in the excessive-Se area and over 78% in the high-Se area (Table S6). This discrepancy can be attributed to the higher soil Se content in the excessive-Se area compared to the high-Se area, which enhances the Se accumulation in the crops cultivated in these regions [2]. In the excessive-Se and high-Se areas, potato had the greatest Se variation coefficient of 125.02% and 140.57%, followed by corn (87.88% and 107.21%), indicating that the Se content of the same crops varied significantly, even when in the same Se-containing area. This was mainly influenced by the total and plant-available Se concentrations in soil, which affect how much Se is absorbed by the crops during growth [2,39]. In many Se-poisoned areas worldwide (e.g., Enshi of Hubei, Ziyang of Shaanxi, Punjab of India, and Abeokuta of Nigeria), Se poisoning has occurred as a result of consuming locally grown crops with elevated Se levels [4,40,41,42]. The mean Se content in corn from the excessive-Se area of this study (0.99 mg/kg) was higher than that of Ziyang (0.81 mg/kg), Enshi (0.41 mg/kg), and Abeokuta (0.10 mg/kg), but was lower than that of Punjab, India (13 mg/kg) [4,40,41,42] The average Se content in corn from the high-Se area (0.30 mg/kg) was only higher than that of Abeokuta (0.10 mg/kg) in Nigeria [41]. The mean Se content in rice from the excessive-Se area of this study (0.43 mg/kg) was lower than that of Ziyang (0.71 mg/kg), Punjab (16.20 mg/kg), and Abeokuta (2.01 mg/kg), but higher than that of Enshi, Hubei Province (0.30 mg/kg) [4,40,41,42]. The mean Se (0.13 mg/kg) in rice from the high-Se area was lower than the levels reported in Ziyang, Enshi, Punjab, and Abeokuta [4,40,41,42]. The mean Se contents of sweet potato from both areas (0.96 mg/kg and 0.89 mg/kg) were higher than that of Enshi (0.36 mg/kg) [43]. The mean Se contents of potato in both areas (0.49 mg/kg and 0.50 mg/kg) were higher than that of Enshi (0.28 mg/kg) and Ziyang (0.37 mg/kg) and were lower than that of Abeokuta (3.06 mg/kg) [4,40,43]. The average Se contents of radish in the areas with excessive Se and high Se levels (1.37 mg/kg and 2.32 mg/kg, respectively) were higher than that in Enshi (0.23 mg/kg) [43]. The average Se contents of eggplant in both areas (0.75 mg/kg and 0.70 mg/kg) were lower those of 3.99 mg/kg in Ziyang and 1.04 mg/kg in Enshi [4,43]. In summary, the Se contents of crops in this study area were high, especially in corn, potato, radish, and sweet potato (Table S7).

### 3.3. Estimated Daily Intake of Se for Humans (Adults and Children) Based on Specific Dietary Combinations

The World Health Organization (WHO) and the Chinese Nutrition Society have established a safe daily Se intake range of 50–400 μg/day for adults [44,45]. While the WHO has not set specific daily Se intake standards for children, the Chinese Nutrition Society has defined daily intake levels for residents of different age groups. In this study, the safe range of daily Se intake for children was determined to be 45–300 μg/day [45]. In the study area, residents mainly follow a low-protein diet with very limited intake of meat, eggs, and dairy. As a result, Se intake is primarily derived from crops and water.

**Figure 3:**
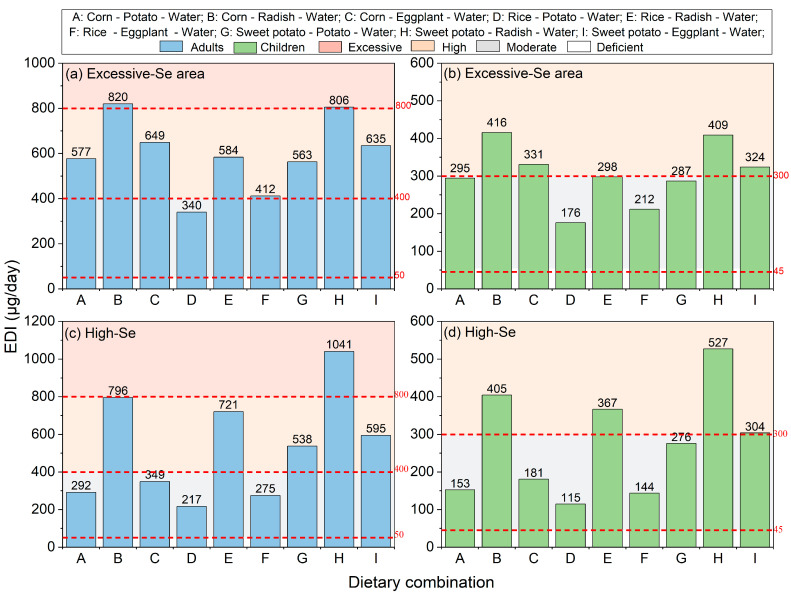
Daily intakes of Se in grain–vegetable–drinking water food combinations (adults and children): (**a**) adults in excessive-Se area; (**b**) children in excessive-Se area; (**c**) adults in high-Se area; (**d**) children in high-Se area.

The average daily Se intakes from excessive-Se areas were 598 μg/day in adults and 305 μg/day in children (Figure 3 and Table S8). Based on different dietary combinations, the estimated average daily Se intake for adults ranged from 340 μg/day to 820 μg/day, with specific values as follows: corn–radish–water (820 μg/day), sweet potato–radish–water (806 μg/day), corn–eggplant–water (649 μg/day), sweet potato–eggplant–water (635 μg/day), rice–radish–water (584 μg/day), corn–potato–water (577 μg/day), sweet potato–potato–water (563 μg/day), rice–eggplant–water (412 μg/day), and rice–potato–water (340 μg/day) (Table S8). Only the dietary combination of rice–potato–water resulted in a total daily Se intake within the safe consumption range, while the remaining eight dietary combinations of food were all higher than the 400 μg/day level that is considered safe for adults, as set by the Chinese Nutrition Society and the WHO [44,45]. Notably, the combinations of corn–radish–water and sweet potato–radish–water even surpassed the marginal upper intake level of 800 μg/day set by the WHO [44,45]. The daily intakes of Se from all crop combinations in the area of this study with excessive Se were lower than the average daily intake of Se for adults in Shuang’an Township, Ziyang County, Shaanxi (1067 μg/day) and in Enshi, Hubei (959 μg/day) [2,4]. In addition, the estimated average daily Se intake for children ranged from 176 μg/day to 416 μg/day, with specific values as follows: corn–radish–water (416 μg/day), sweet potato–radish–water (409 μg/day), corn–eggplant–water (331 μg/day), sweet potato–eggplant–water (324 μg/day), rice–radish–water (298 μg/day), corn–potato–water (295 μg/day), sweet potato–potato–water (287 μg/day), rice–eggplant–water (212 μg/day), and rice–potato–water (176 μg/day) (Table S8). The dietary combinations of rice–potato–water, rice–radish–water, rice–eggplant–water, sweet potato–potato–water, and corn–potato–water resulted in a total daily Se intake within the safe consumption range, while the remaining four dietary combinations exceeded the tolerable upper intake of 300 μg/day for children, as stipulated by the Chinese Nutrition Society [45]. In summary, the safe dietary combination from the excessive-Se area for adults was rice–potato–water, and for children were rice–potato–water, rice–radish–water, rice–eggplant–water, sweet potato–potato–water and corn–potato–water.

The average daily Se intakes from high-Se areas were 536 μg/day in adults and 275 μg/day in children (Figure 3 and Table S8). The estimated average daily Se intake for adults ranged from 217 μg/day to 1041 μg/day, with specific values as follows: sweet potato–radish–water (1041 μg/day), corn–radish–water (796 μg/day), rice–radish–water (721 μg/day), sweet potato–eggplant–water (595 μg/day), sweet potato–potato–water (538 μg/day), corn–eggplant–water (349 μg/day), corn–potato–water (292 μg/day), rice–eggplant–water (275 μg/day), and rice–potato–water (217 μg/day) (Table S8). The dietary combinations of sweet potato–radish–water, corn–radish–water, rice–radish–water, sweet potato–potato–water, and sweet potato–eggplant–water were found to exceed the tolerable upper daily dietary Se intake levels of 400 μg/day for adults, as established by the Chinese Nutrition Society and the WHO. Particularly noteworthy was that the combination of sweet potato–radish–water even surpassed the marginal upper intake level of Se for adults set by the WHO (800 μg/day). This indicates that long-term consumption of most dietary combinations poses a risk of Se poisoning in adults living in areas with high Se levels [44,45]. The daily intakes from all crop combinations exceeded the reported average adult intake of 158 μg/day in Enshi, Hubei Province, China [2]. In addition, the average daily Se intakes of children from different dietary combinations were 527 μg/day (sweet potato–radish–water), 405 μg/day (corn–radish–water), 367 μg/day (rice–radish–water), 304 μg/day (sweet potato–eggplant–water), 276 μg/day (sweet potato–potato–water), 181 μg/day (corn–eggplant–water), 153 μg/day (corn–potato–water), 144 μg/day (rice–eggplant–water), and 115 μg/day (rice–potato–water) (Table S8). The dietary combinations of total daily Se intakes of sweet potato–radish–water, corn–radish–water, rice–radish–water, and sweet potato–eggplant–water exceeded the tolerable upper daily dietary Se intake level for children, as established by the Chinese Nutrition Society (300 μg/day) [45]. In summary, the safe dietary combinations from high-Se areas for adults and children were corn–potato–water, rice–potato–water, corn–eggplant–water, and rice–eggplant–water.

Different countries and agencies around the world have established dietary intake standards for Se, including upper intake levels and recommended intake levels [46]. For example, the Institute of Medicine Food and Nutrition Board (IMFNB) in the United States recommends Se intake levels of 55 μg/day for adults and 40 μg/day for children, with safe upper levels set at 400 μg/day and 280 μg/day, respectively [47]. Meanwhile, the European Food Safety Authority (EFSA) recommends Se intake levels of 70 μg/day for adults and 55 μg/day for children, with safe upper levels of 300 μg/day for adults and 200 μg/day for children [48,49]. In the excessive-Se area of this study, the Se intake levels for adults (340–820 μg/day) and children (176–416 μg/day) across all dietary combinations (except the dietary combination of rice–potato–water) exceeded the safe upper levels set by the EFSA (300 μg/day for adults and 200 μg/day for children). Except for the dietary combinations of rice–potato–water (340 μg/day for adults and 176 μg/day for children) and rice–eggplant–water (212 μg/day for children), all other combinations in the excessive-Se area had adult (400 μg/day) and child (280 μg/day) intake levels exceeding the safe upper levels established by the IMFNB. Furthermore, in the high-Se area, the dietary combinations of corn–potato–water (292 μg/day), rice–potato–water (217 μg/day), and rice–eggplant–water (275 μg/day) showed adult intake levels below the safe upper limits set by the IMFNB (400 μg/day) and the EFSA (300 μg/day). The dietary combinations of corn–potato–water (153 μg/day), rice–eggplant–water (181 μg/day), rice–potato–water (115 μg/day), and rice–eggplant–water (144 μg/day) had children’s intake levels below the safe upper levels set by the IMFNB (280 μg/day) and the EFSA (200 μg/day). Overall, the Se intake levels across all dietary combinations in both the excessive-Se and high-Se areas exceeded the recommended Se intake levels set by the EFSA and the IMFNB [47,48,49].

### 3.4. Estimated Daily Intake of Se for Humans (Adults and Children) Based on Diet Dietary Diversity Combination

To gain a more comprehensive understanding of dietary intake based on daily dietary diversity among adults and children in the excessive-Se and high-Se areas, this study maintains the proportion of grain, vegetable, and drinking water intake while adjusting the proportions of grains and vegetables. Specifically, the ratio of rice-corn-sweet potato is set at 4:3:3, and the ratio of potatoes-radishes-eggplants is also 4:3:3 (Table S9). In the excessive-Se area, the estimated average daily Se intake was 572 μg/day for adults and 292 μg/day for children. In contrast, the average daily Se intake for adults and children in high-Se areas is 503 μg/day and 258 μg/day, respectively. Therefore, Se intake for adults exceeds the WHO and Chinese Nutrition Society’s limit of 400 μg/day, while children’s intake remains within a safe range [44,45]. Furthermore, the average daily Se intake based on a diet diversity combination is slightly lower than that based on specific dietary combinations.

**Figure 4:**
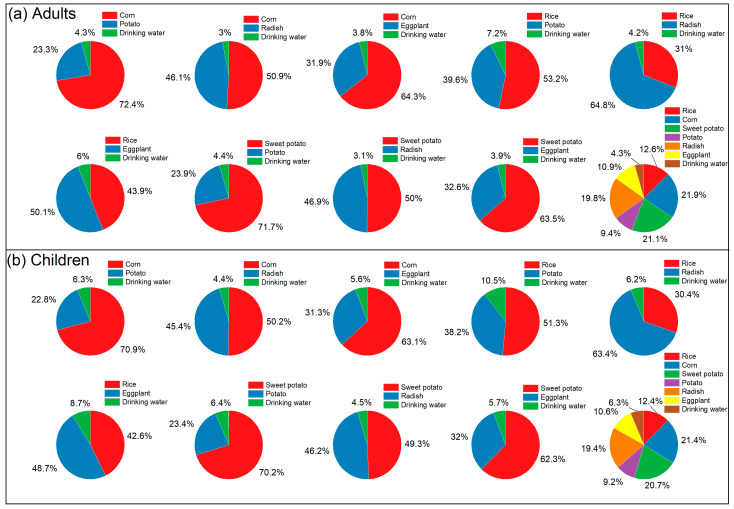
Daily Se intake contribution for residents in the excessive-Se area: (**a**) adults in excessive-Se area; (**b**) children in excessive-Se area.

**Figure 5:**
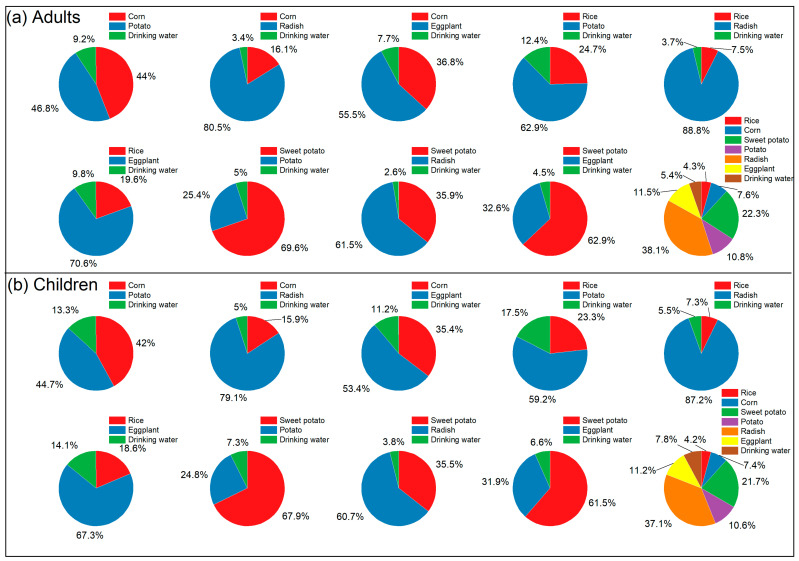
Daily Se intake contribution for residents in the high-Se area: (**a**) adults in high-Se area; (**b**) children in high-Se area.

By analyzing the dietary Se intake of adults and children in excessive-Se and high-Se areas, targeted recommendations can be made to improve Se nutrition for local residents, thereby reducing the risk of excessive Se intake. As shown in Figure 4 and Figure 5, grains and vegetables constitute the main dietary sources of Se in the studied excessive-Se and high-Se areas. In the excessive-Se area, over 90% of dietary Se comes from grains and vegetables (Figure 4), while in the high-Se area, more than 80% of dietary Se comes from grains and vegetables (Figure 5). A comparison between dietary combinations based on dietary diversity and specific dietary combinations reveals that dietary diversity can alter the relative contribution of Se from crops. This approach helps to reduce the daily intake of high-Se crops, which is crucial for safe Se supplementation (Figure 4 and Figure 5).

Drinking water is another way of Se supplementation in the study area. In both the excessive-Se and high-Se areas, the contribution of drinking water to the diet of children is higher than that of adults. Additionally, the proportional contribution of Se in the diets from drinking water of residents in excessive-Se area is lower compared to that in the high-Se area (Figure 4 and Figure 5). Specifically, the average proportion of drinking water in the diets of adults in the excessive-Se area is 4.43%, while for children, it is 6.48%. In the high-Se area, the average proportion of drinking water in adult diets is 6.50%, and for children, it is 9.38%. Therefore, in both excessive-Se and high-Se areas, the supplementation of Se through drinking water for children should not be underestimated.

### 3.5. Comparison of Daily Intake Between the Study Area and Se-Deficient Areas

**Figure 6:**
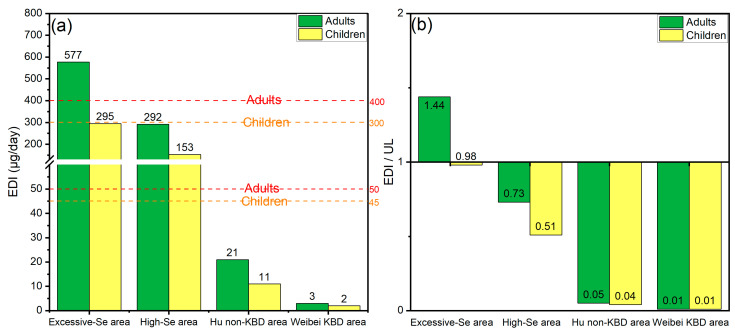
Daily intake of residents in different areas (**a**) and the ratio of daily intake to the daily intake upper limit for residents in different areas (**b**).

The daily intake of residents in the study area was compared with those from typical Se-deficient areas. The KBD area was recognized as a typical Se-deficient area, highlighting the need for residents to incorporate Se-rich foods into their diet [3]. The prevalence of KBD was closely related to environmental Se deficiency, with Se deficiency considered one of the contributing factors to KBD [50]. Zhang et al. (2022) found that Se deficiency in the diet is still an important factor inducing KBD by studying the element content in crops and drinking water from KBD and non-KBD areas in the Shaanxi Province [3]. Therefore, it is imperative for residents of the KBD area to supplement Se through dietary sources. To clarify the differences in Se intake between the residents of this study area and those of other typical Se-containing areas, the daily Se intake of residents from Se-rich (excessive-Se area and high-Se area) and Se-deficient areas (KBD area and non-KBD area in Shaanxi Province) was compared in this study (Figure 6 and Table S10).

Corn, potatoes, and drinking water are common crops in the study area (Se-rich areas including excessive-Se and high-Se areas) and the Se-deficient areas (KBD and non-KBD areas) in the Shaanxi Province. Therefore, we selected the combination of corn–potato–drinking water as the dietary group for the intake comparison studies. The mean Se content in corn from the excessive-Se area was 992.07 μg/kg, which was 23.61 times and 251.79 times of that in the Hu non-KBD area (non-KBD area of Hu County) (42.02 μg/kg) and the Weibei KBD area (KBD area of north in Weihe River) (3.94 μg/kg), respectively. The mean Se content in corn from the high-Se area was 304.80 μg/kg, which was 7.25 times and 77.36 times of that in the Hu non-KBD and Weibei KBD areas, respectively. The average Se content of potato in the excessive-Se area was 487.71 μg/kg, which was 40.98 times and 1806.33 times of that in the Hu non-KBD (11.90 μg/kg) and the Weibei KBD (0.27 μg/kg) areas, respectively. The mean Se content in potato from the high-Se area was 495.11 μg/kg, which was 41.61 times and 1833.74 times of that in Hu non-KBD area and Weibei KBD area, respectively. The average Se content of drinking water in the excessive-Se area was 12.32 μg/L, which was 58.67 times and 28 times of that in the Hu non-KBD (0.21 μg/L) and Weibei KBD (0.44 μg/L) areas, respectively. The average Se content of drinking water in the high-Se area was 13.50 μg/L, which was 64.29 times and 30.68 times of that in the Hu non-KBD and Weibei KBD areas, respectively. The daily intakes of adults (children) from excessive-Se and high-Se areas were 577 μg/day (295 μg/day) and 292 μg/day (153 μg/day), respectively (Table S10).

The daily Se intakes of adults (children) in the Hu non-KBD area and Weibei KBD area were 21 μg/day (11 μg/day) and 3 μg/day (2 μg/day), respectively. Surprisingly, the daily intakes of adults (children) from excessive-Se and high-Se areas were 26.97 (27.28) times and 13.65 (14.14) times higher than those in the Hu non-KBD area, respectively. The daily intakes of adults (children) from excessive-Se and high-Se areas were 221.04 (192.56) times and 111.87 (99.82) times higher than those in the Weibei KBD area, respectively. The WHO and China Nutrition Society have stipulated that the safe range of daily Se intake for adults and children is 50–400 μg/day and 45–300 μg/day, respectively [44,45]. Despite that Se intakes in the Hu non-KBD and Weibei KBD areas were far below the lower limit of the safe daily intake range, there was no KBD in the Hu non-KBD area [3]. Therefore, the daily intake of Se for adults (21 μg/day) and children (11 μg/day) was considered acceptable. This was similar to the 20 μg/day recommended intake for the prevention of KBD or KD in endemic areas [51]. It is worth noting that the daily intakes of Se for adults and children from the excessive-Se area and high-Se area were 1.44 (0.98) and 0.73 (0.51) times the tolerable upper limit of safe intake, respectively, while the daily intake of Se for adults (and children) in the Hu non-KBD area and Weibei KBD area was only 0.05 (0.04) and 0.01 (0.01) times the tolerable upper limit of safe intake, respectively. Therefore, it is economically and practically significant to sell agricultural products from excessive-Se areas and high-Se areas to low-Se areas (especially KBD areas).

### 3.6. Health Risk Assessment of Humans (Adults and Children) in Excessive-Se and High-Se Areas

**Figure 7:**
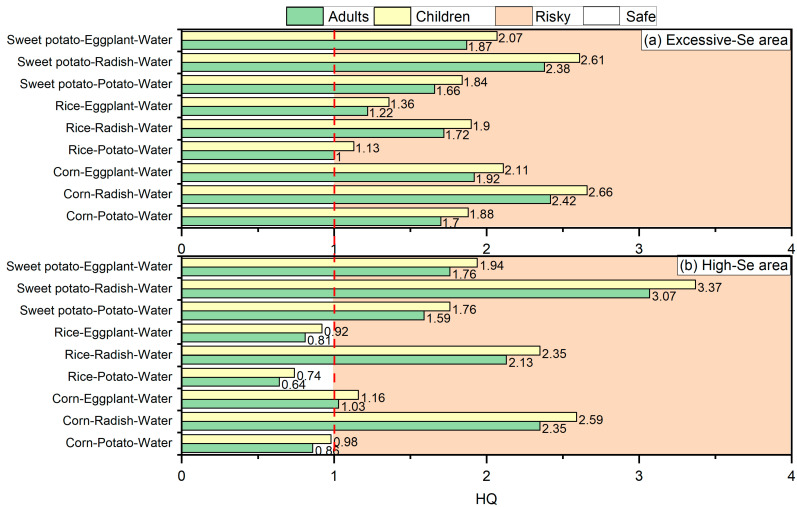
Hazard quotient (HQ) of Se in grain–vegetable–drinking water food combinations (adults and children): (**a**) excessive-Se area; (**b**): high-Se area.

To evaluate the possible health risks associated with the long-term consumption of local crops and drinking water by adults and children in the excessive-Se and high-Se areas of northern Langao County, this study assessed the non-carcinogenic risks of adults and children through nine different dietary combinations of grains, vegetables, and drinking water (Figure 7 and Table S11).

The average HQ of adults (children) from the excessive-Se area was 1.77 (1.95) (Table S11). It was found that the HQs of Se for adults ingesting dietary combinations from the excessive-Se area were in the order of 2.42 (corn–radish–water), 2.38 (sweet potato–radish–water), 1.92 (corn–eggplant–water), 1.87 (sweet potato–eggplant–water), 1.70 (corn–potato–water), 1.66 (sweet potato–potato–water), 1.72 (rice–radish–water), 1.22 (rice–eggplant–water), and 1.00 (rice–potato–water), which were all lower than the HQ (4.34) for adults in Shuang’an of Ziyang County, but are all values higher than 1. Therefore, there might be potential non-carcinogenic risk associated with long-term intake of the nine dietary combinations [4,28]. The HQs of Se for children ingesting dietary combinations from the excessive-Se area were in the order of 2.66 (corn–radish–water), 2.61 (sweet potato–radish–water), 2.11 (corn–eggplant–water), 2.07 (sweet potato–eggplant–water), 1.90 (rice–radish–water), 1.88 (corn–potato–water), 1.84 (sweet potato–potato–water), 1.36 (rice–eggplant–water), and 1.13 (rice–potato–water), which were all higher than 1, indicating that long-term consumption of these dietary combinations may have potential non-carcinogenic risks. Adults and children from excessive-Se areas are exposed to potential non-carcinogenic risks through the consumption of the nine dietary combinations, and the non-carcinogenic risk is higher in children than in adults.

The average HQ of adults (and children) from the high-Se area was 1.58 (1.76) (Table S11). The HQs of Se in adult dietary combinations from high-Se areas were 3.07 (sweet potato–radish–water), 2.35 (corn–radish–water), 2.13 (rice–radish–water), 1.76 (sweet potato–eggplant–water), 1.59 (sweet potato–potato–water), 1.03 (corn–eggplant–water), 0.86 (corn–potato–water), 0.81 (rice–eggplant–water), and 0.64 (rice–potato–water). The HQs of Se in six dietary combinations (sweet potato–radish–water, corn–radish–water, rice–radish–water, sweet potato–potato–water, sweet potato–eggplant–water and corn–eggplant–water) were greater than one, indicating that the long-term consumption of these dietary combinations may have potential non-carcinogenic risks. The HQs of Se in children’s dietary combinations from the high-Se areas were 3.37 (sweet potato–radish–water), 2.59 (corn–radish–water), 2.35 (rice–radish–water), 1.94 (sweet potato–eggplant–water), 1.76 (sweet potato–potato–water), 1.16 (corn–eggplant–water), 0.98 (corn–potato–water), 0.92 (rice–eggplant–water), and 0.74 (rice–potato–water). The HQs of Se in six dietary combinations (sweet potato–radish–water, corn–radish–water, rice–radish–water, sweet potato–potato–water, sweet potato–eggplant–water and corn–eggplant–water) were greater than one, indicating that the long-term consumption of dietary combinations may have potential non-carcinogenic risk. Adults and children in high-Se areas face potential non-carcinogenic risk from consuming the six dietary combinations assessed, with children exhibiting higher non-carcinogenic risk levels than adults. It should be noted that this study was limited to evaluating health risks through the combination of crops and drinking water. It is important to note that daily exposure to Se can also occur through other pathways, such as soil and air. It is therefore recommended that residents in excessive-Se and high-Se areas diversify their dietary structure by incorporating a greater variety of dietary products from low-Se regions, which could help reduce the intake of potentially harmful dietary combinations.

In addition, this study has several limitations. First, the health-based reference values employed, including the RfD for non-cancer risk and the clinical threshold for selenosis, are primarily derived from epidemiological studies on adult populations. Applying these thresholds directly to assess risks for children introduces uncertainty, as children may have a different Se metabolism, higher intake per body weight, and potentially greater susceptibility. In the absence of region-specific and age group-specific guidance values, the use of adult-derived benchmarks represents a common and conservative approach in exposure assessment. It provides a preliminary screening tool, but the results should be interpreted with caution for younger subgroups. In addition, a limitation of this study is the absence of Se speciation data. Future research should conduct detailed speciation analysis to accurately assess health risks.

## 4. Conclusions

This study investigated the Se content, estimated daily intake, and health risk for humans from high-Se areas and excessive-Se areas using the grain–vegetable–water system. The average Se content of drinking water in excessive-Se (12.32 μg/L) and high-Se (13.50 μg/L) areas met China’s mineral water standards (10–50 μg/L), and the Se enrichment rate exceeds 60%. The crops with the highest average Se content were corn (0.99 mg/kg), rice (0.43 mg/kg), sweet potato (0.96 mg/kg), and eggplant (0.75 mg/kg) in the area with excessive Se levels, and potato (0.50 mg/kg) and radish (2.32 mg/kg) in the area with high Se levels, exhibited the highest average Se content among all crops. The Se content of rhizome (radish) was higher than that of grain (rice), and all crops showed Se-enrichment rates exceeding 78%. The average daily intakes of Se in adults and children from the excessive-Se area were 598 μg/day and 305 μg/day, respectively, and the safe dietary combination from the excessive-Se area for adults was rice–potato–water, and for children were rice–potato–water, rice–radish–water, rice–eggplant–water, sweet potato–potato–water, and corn–potato–water. The estimated average daily Se intake in the high-Se area was 536 μg/day for adults and 275 μg/day for children, and the safe dietary combinations for both adults and children were corn–potato–water, rice–potato–water, corn–eggplant–water, and rice–eggplant–water. Residents who have dietary Se that comes from crops are over 90% in the excessive-Se area and 80% in the high-Se area; crops are the primary sources of dietary Se in these two areas. However, the average number of adults (and children) residing in areas of excessive-Se and high-Se with their dietary average Se coming from drinking water are 4.43 (6.48) % and 6.50 (9.38) %, respectively; the supplementation of Se through drinking water for children should not be underestimated. The average HQs of adults (children) from excessive-Se and high-Se areas were 1.77 (1.95) and 1.58 (1.76), respectively, and residents living in these two areas possess non-carcinogenic risks (HQ > 1). Additionally, the non-carcinogenic risks of children in both excessive-Se and high-Se areas were higher than those of adults. Potential non-carcinogenic risks (HQ > 1) of adults (and children) from excessive-Se areas consuming all dietary combinations, and from high-Se areas eating dietary combinations including corn/rice/sweet potato–radish–water, sweet potato–potato/eggplant–water, and corn–eggplant–water. The dietary items in high-Se areas and excessive-Se areas are good dietary Se sources in low-Se areas.

This study provides insights into the maximum health tolerance of residents in excessive-Se and high-Se areas under specific dietary patterns. However, this study still has some limitations. A better understanding of dietary Se intake and its health risks across populations in the study area requires more detailed biomarker studies and dose–response assessments, particularly in sensitive groups like children.

## Figures and Tables

**Table 1:** Definition and reference value of some parameters for health risk assessment [28,29].

Factor	Definition	Unit	Adult	Children
EF	Exposure frequency	day/year	350	350
ED	Exposure duration	years	30	12
BW	Body weight	kg	65	30
AT	Average time	day	10,950	4380
RfD	Reference dose	mg/kg/day	0.005	0.005
EDI	Estimated daily intake	mg/day	-	-
CDI	Chronic daily intake	mg/kg/day	-	-

“-” No data.

## Data Availability

The original contributions presented in the study are included in the article/Supplementary Materials. Further inquiries can be directed to the corresponding authors.

## Supplementary Materials

The following supporting information can be downloaded at: https://www.mdpi.com/article/10.3390/foods15010031/s1, Table S1: The operating parameter for ICP-MS/MS instrument; Table S2: The operating parameter for HG-AFS instrument; Table S3: Comparison of experiment values and certified values of the GBW10010a (rice) and GBW10012 (corn); Table S4: Quality control for determining the Se concentrations in crop and water; Table S5: Se content in water samples; Table S6: Results statistics of Se content in crops; Table S7: Comparison of Se content of crops in this study with other selenosis regions; Table S8: Estimated daily intake (EDI) of total Se for adults and children in food combinations in different Se-containing areas; Table S9: The estimated daily intake (EDI) of Se through all main routes for adults and children in excessive-Se area and high-Se area; Table S10: Estimated daily intake (EDI) of residents in different areas and the ratio of daily intake to the daily intake upper limit for residents in different areas; Table S11. Hazard quotient (HQ) of Se in food combinations of grain-vegetable-drinking water (adults and children); References [4,31,32,33,36,40,41,42,43] are cited in the supplementary materials.
