# Supplementary material for: Selenium Content in Staple Crops and Drinking Water and Associated Health Risk Assessment: A Case Study of a Selenium-Rich Region in Southern Shaanxi, China
Source: Foods. 2025 Dec 22;15(1):31. doi: 10.3390/foods15010031 (PMC12785991; doi:10.3390/foods15010031)
Supplement: Supplementary file 1 [file foods-15-00031-s001.zip › foods-4016351-supplementary.pdf]

Supplementary Files

Table S1. The operating parameter for ICP-MS/MS instrument

Parameter	Value
Scan mode	MS/MS
RF power (W)	1550
Sampling depth (mm)	9
Nebulizer gas flow (L/min)	0.7
Make-up gas flow (L/min)	0.4
Extract 1 (V)	0
Extract 1 (V)	-180
Cell gas flow (mL/min)	O_2=0.4
Q1/Q2	78/94 (Se)

Table S2. The operating parameter for HG-AFS instrument

Parameter	Value
Negative high voltage (V)	280
Lamp current (mA)	80
Atomizer height (mm)	8
Atomizer temperature (°C)	200
Carrier gas flow (mL/min)	400
Shielded gas flow (mL/min)	800
Reading duration (s)	7.0
Delay time (s)	1.5

Table S3. Comparison of experiment values and certified values of the GBW10010a (rice) and GBW10012 (corn)

Analyte	GBW10010a (rice)			GBW10012 (corn)		
	Certified value (mg/kg)	Measured value (mg/kg)	Recovery (%)	Certified value	Measured value (mg/kg)	Recovery (%)
Se	0.036±0.008	0.038	105.6	0.021±0.008	0.023	109.5
		0.035	97.2		0.019	90.5

Table S4. Quality control for determining the Se concentrations in crop and water

Element	Calibration range (μg/L)	Calibration Equation	R^2	Detection limit (μg/L)	Quantification limit (μg/L)
Se-Crop	0-20	I = 98.4548*C + 18.5056	0.9999	0.036	0.12
Se-Water	0-50	C / S = (228.4 + 1010*conc)*[I/S Ratio]	0.9999	0.003	0.01

Table S5. Se content in water samples

Items	Excessive-Se (>3 mg/kg)	High-Se (0.4-3 mg/kg)
Number of total samples	23	16
Maximum (μg/L)	30.52	41.19
Minimum (μg/L)	0.31	0.55
Average (μg/L)	12.32	13.50
Coefficient of Variation (%)	57.08	86.75
Number of samples (Se⩾10μg/L)	16	10
Se-enriched rate (%)	69.57	62.5
CNS[31] (μg/L)	⩽10.00	⩽10.00
INS[32] (μg/L)	⩽40.00	⩽40.00
CNSM[33] (μg/L)	10-50	10-50

Note:“CNS” Chinese national standard for drinking water quality; “INS” international guidelines for drinking water quality; “CNSM” Chinese national standard for drinking natural mineral water quality

Table S6. Results statistics of Se content in crops

Catalog	Excessive-Se (>3 mg/kg)						High-Se (0.4-3 mg/kg)					
	Grains			Vegetables			Grains			Vegetables		
	Corn	Rice	Sweet potato	Potato	Radish	Eggplant	Corn	Rice	Sweet potato	Potato	Radish	Eggplant
Number	20	7	6	10	7	6	14	5	6	8	3	5
Max (mg/kg)	2.82	0.44	0.97	1.77	2.45	0.77	1.06	0.14	1.07	1.78	4.28	0.75
Min (mg/kg)	0.08	0.42	0.94	0.04	1.10	0.73	0.02	0.11	0.74	0.04	1.18	0.65
Ave (mg/kg)	0.99	0.43	0.96	0.49	1.37	0.75	0.30	0.13	0.89	0.50	2.32	0.70
CV (%)	87.88	1.47	0.97	125.02	32.40	2.03	107.21	8.13	14.67	140.57	60.07	4.74
Rate (%)	100	100	100	100	100	100	78.57	100	100	100	100	100
Sta[36] (mg/kg)	≥0.05	≥0.05	≥0.05	≥0.02	≥0.02	≥0.02	≥0.05	≥0.05	≥0.05	≥0.02	≥0.02	≥0.02

Note: "Max" Maximum; "Min" Minimum; "Ave" Average; "CV" Coefficient of variation; "Rate" Se-enriched rate; "Std" Se-enriched standard;

Table S7. Comparison of Se content of crops in this study with other selenosis regions

Crop	Region	Se content (mg/kg)	Reference
Corn	Excessive-Se	0.99	This study
	High-Se	0.30	This study
	Ziyang, China	0.81	[4]
	Enshi, China	0.41	[42]
	Abeokuta, Nigeria	0.10	[40]
	Punjab, India	13.00	[41]
Rice	Excessive-Se	0.43	This study
	High-Se	0.13	This study
	Ziyang, China	0.71	[4]
	Enshi, China	0.30	[42]
	Abeokuta, Nigeria	2.01	[40]
	Punjab, India	16.20	[41]
Sweet potato	Excessive-Se	0.96	This study
	High-Se	0.89	This study
	Enshi, China	0.36	[43]
Potato	Excessive-Se	0.49	This study
	High-Se	0.50	This study
	Ziyang, China	0.37	[4]
	Enshi, China	0.28	[43]
	Abeokuta, Nigeria	3.06	[40]
Radish	Excessive-Se	1.37	This study
	High-Se	2.32	This study
	Enshi, China	0.23	[43]
Eggplant	Excessive-Se	0.75	This study
	High-Se	0.70	This study
	Ziyang, China	3.99	[4]
	Enshi, China	1.04	[43]

Table S8. Estimated daily intake (EDI) of total Se for adults and children in food combinations in different Se-containing areas

Food combinations	Resident	EDI (μg/day)	
		Excessive-Se area	High-Se area
corn - potato - water	Adults	577	292
	Children	295	153
corn - radish - water	Adults	820	796
	Children	416	405
corn - eggplant - water	Adults	649	349
	Children	331	181
rice - potato - water	Adults	340	217
	Children	176	115
rice - radish - water	Adults	584	721
	Children	298	367

rice - eggplant - water	Adults	412	275
	Children	212	144
sweet potato - potato - water	Adults	563	538
	Children	287	276
sweet potato - radish - water	Adults	806	1041
	Children	409	527
sweet potato - eggplant - water	Adults	635	595
	Children	324	304
Average value	Adults	598	536
	Children	305	275

Table S9. The estimated daily intake (EDI) of Se through all main routes for adults and children in excessive-Se area and high-Se area

Crop	Intake rate (g/day or L/day)		Average Se concentration (mg/kg or mg/L)		EDI (μg/day)			
					Excessive-Se		High-Se	
	Adults	Children	Excessive-Se	High-Se	Adults	Children	Adults	Chindren
Rice	168.40	84.20	0.43	0.13	72	36	21	11
Corn	126.30	63.15	0.99	0.30	125	63	38	19
Sweet potato	126.30	63.15	0.96	0.89	121	60	112	56
Potato	110.40	55.20	0.49	0.50	54	27	55	27
Radish	82.80	41.40	1.37	2.32	113	57	192	96
Eggplant	82.80	41.40	0.75	0.70	62	31	58	29
Water	2	1.5	0.01	0.01	25	18	27	20
Total	-	-	-	-	572	292	503	258

Note: "-"No data; Intake ratio of Grains (Rice : Corn : Sweet potato) = 4 : 3 : 3; Intake ratio of vegetables (Potato : Radish : Eggplant) = 4 : 3 : 3.

Table S10. Estimated daily intake (EDI) of residents in different areas and the ratio of daily intake to the daily intake upper limit for residents in different areas

Area	Average Se content			EDI (μg/day)		EDI / UL	
	Corn (μg/kg)	Potato (μg/kg)	Drinking water (μg/L)	Adult	Children	Adult	Children
Excessive-Se area	992.07	487.71	12.32	576.91	294.61	1.44	0.98
High-Se area	304.80	495.11	13.50	291.97	152.73	0.73	0.51
Hu non-KBD area	42.02	11.90	0.21	21.39	10.80	0.05	0.04
Weibei KBD area	3.94	0.27	0.44	2.61	1.53	0.01	0.01

Note: "EDI/UL" Estimated daily intake / Upper limit.

Table S11. Hazard quotient (HQ) of Se in food combinations of grain-vegetable-drinking water (adults and children)

Food combinations	Excessive-Se		High-Se	
	Adult	Children	Adult	Children
Corn-Potato-Water	1.70	1.88	0.86	0.98
Corn-Radish-Water	2.42	2.66	2.35	2.59
Corn-Eggplant-Water	1.92	2.11	1.03	1.16
Rice-Potato-Water	1.00	1.13	0.64	0.74
Rice-Radish-Water	1.72	1.90	2.13	2.35
Rice-Eggplant-Water	1.22	1.36	0.81	0.92
Sweet potato-Potato-Water	1.66	1.84	1.59	1.76
Sweet potato-Radish-Water	2.38	2.61	3.07	3.37
Sweet potato-Eggplant-Water	1.87	2.07	1.76	1.94
Average value	1.77	1.95	1.58	1.76
